# Epigenetic modifications in cancer drug resistance: molecular mechanisms and therapeutic interventions

**DOI:** 10.1186/s43556-026-00458-9

**Published:** 2026-04-29

**Authors:** Jingyi Yang, Minpu Zhang, Yuting Zhong, Changgang Sun, Jing Zhuang

**Affiliations:** 1 College of First Clinical Medicine, Shandong University of Traditional Chinese Medicine, Jinan, 250014 China; 2 Faculty of Chinese Medicine, Macau University of Science and Technology, Macau, 999078 China; 3 School of Integrative Medicine, Shanghai University of Traditional Chinese Medicine, Shanghai, 201203 China; 4 College of Traditional Chinese Medicine, Shandong Second Medical University, Weifang, 261000 China; 5 Department of Oncology, Weifang Traditional Chinese Hospital, Weifang, 261000 China

**Keywords:** Cancer therapy resistance, Epigenetic reprogramming, Epigenetic drugs, Combinatorial therapy

## Abstract

Therapeutic resistance remains a major cause of treatment failure and disease recurrence across cancer types, considerably limiting the long-term efficacy of chemotherapies, targeted therapies, and immunotherapies. Growing evidence indicates that resistance cannot be fully explained by static genetic alterations but rather arises from dynamic and reversible adaptive processes. Epigenetic regulation governs transcriptional plasticity, cellular state transitions, and tumor heterogeneity under therapeutic stress. Alterations in DNA methylation, histone modifications, chromatin accessibility, and non-coding RNA networks enable cancer cells to silence tumor suppressor programs, activate compensatory survival pathways, acquire stem cell-like drug-tolerant persister states, and remodel the tumor immune microenvironment. These mechanisms often act in a coordinated manner to form a dynamic regulatory system that supports adaptive resistance. However, current studies have frequently focused on individual epigenetic regulators and have lacked an integrated framework to explain how epigenetic plasticity collectively drives therapeutic resistance. In this review, we deconstruct cancer therapy resistance using the conceptual framework of the “epigenetic landscape.” We summarize the molecular functions and crosstalk among the major epigenetic layers and describe how this integrated network sustains key resistance-associated phenotypes. We also discuss emerging therapeutic strategies that target epigenetic plasticity, including epigenetic drugs, targeted protein degradation, epigenetic editing, and rational combination therapies. Overall, this review provides a systematic framework for understanding epigenetically mediated therapy resistance and highlights epigenetic plasticity as a therapeutic vulnerability for developing durable cancer treatments.

## Introduction

Over the century-long fight against cancer, the emergence and progression of therapeutic resistance have remained one of the most formidable obstacles to durable clinical benefits and long-term survival [1]. For decades, the prevailing models have been shaped by the paradigm of genetic determinism, which attributes acquired resistance primarily to fixed genomic alterations arising from stochastic DNA mutations [2, 3]. Under drug-induced selective pressure, these mutations can confer survival advantages by directly altering drug targets, amplifying oncogenic drivers to activate bypass pathways, and increasing the expression of drug efflux transporters [4]. Despite its influence, this classical model fails to fully account for the complexity and dynamics of resistance evolution [4, 5]. However, several critical questions remain: How are resistant clones selected and expanded within tumor populations that share near-identical genetic backgrounds? [6, 7] Why do certain resistant states persist without stable genetic alterations and even revert after treatment withdrawal? [8] These observations strongly indicate that a regulatory layer beyond the DNA sequence, which is inherently dynamic and plastic, plays a decisive role in the evolution of adaptive tumors. This realization has shifted attention toward epigenetic mechanisms as central drivers of therapeutic escape [9].

**Fig. 1:**
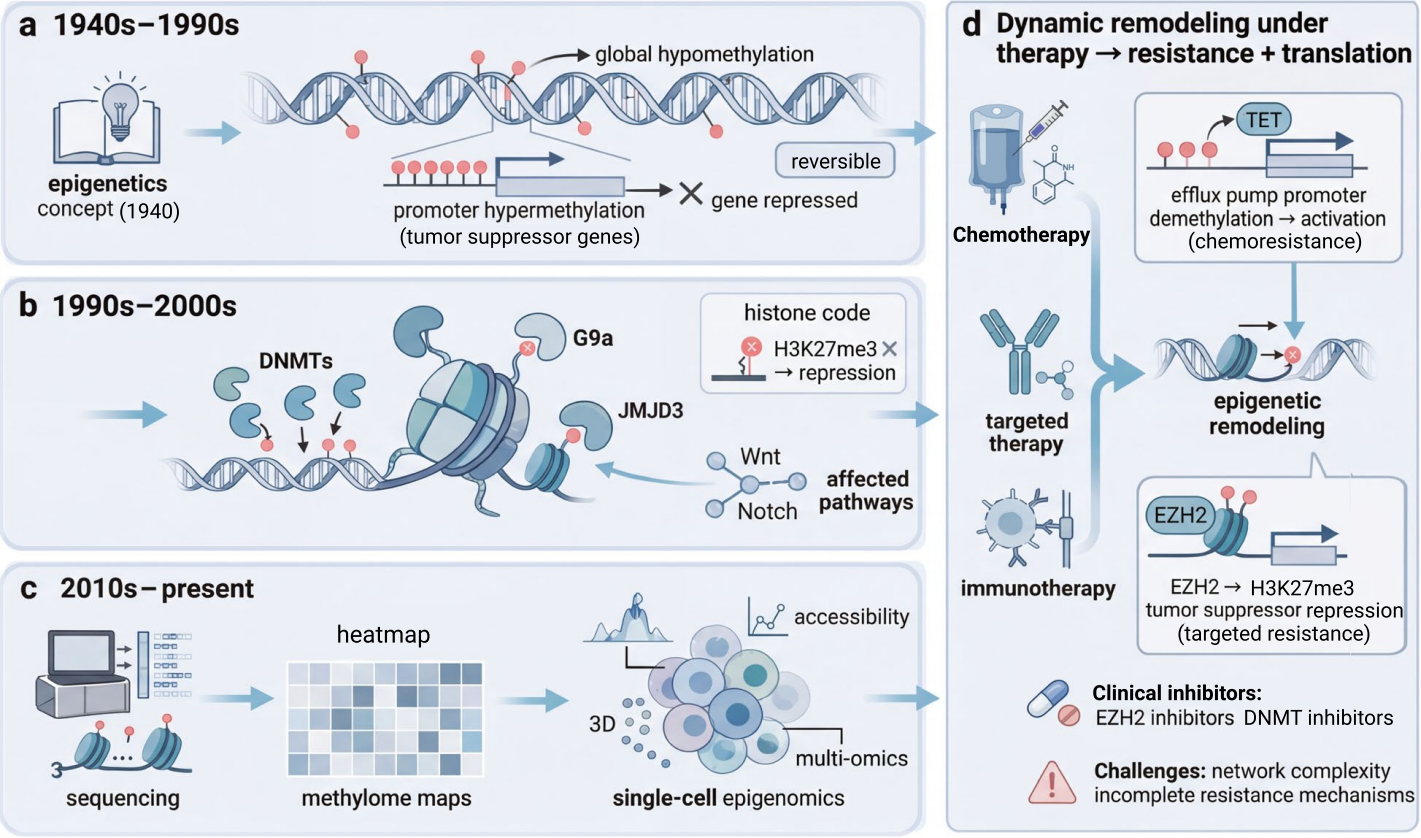
Evolution of tumor epigenetics and mechanisms of resistance. **a** 1940s‒1990s: Conrad Waddington’s metaphor of the “epigenetic landscape” illustrated the principles of cellular fate determination. Subsequent research identified global DNA hypomethylation and focal promoter hypermethylation of tumor suppressor genes as hallmarks of cancer. **b** 1990s‒2000s: The discovery of “writer” and “eraser” enzymes, together with the “histone code” hypothesis, established the molecular basis for reversible epigenetic regulation. **c** 2010s‒present: High-throughput technologies enabled comprehensive mapping of the tumor epigenome, revealing dynamic reprogramming under treatment pressure. **d** Dynamic remodeling and resistance: Treatment-induced epigenetic modifications lead to reactivation or silencing of key genes, driving resistance

The field of epigenetics has developed in parallel with the expanding understanding of tumor complexity. In the 1980 s, researchers observed the coexistence of global hypomethylation and promoter hypermethylation of specific genes in cancer cells, thereby linking epigenetic dysregulation to tumorigenesis [10]. In the 1990 s, the identification and functional characterization of DNA methyltransferases (DNMTs) and histone-modifying enzymes led to the formulation of a histone code hypothesis, providing a conceptual framework for chromatin-based gene regulation [11]. In the twenty-first century, particularly after 2010, advances in high-throughput sequencing technologies enabled genome-wide profiling of epigenetic landscapes, revealing their dynamic remodeling in response to diverse therapeutic pressures [12]. Accumulating evidence indicates that chemotherapy, targeted therapy, and immunotherapy induce extensive epigenetic reprogramming of tumor cells. For example, cytotoxic chemotherapy can remodel promoter-associated chromatin states, leading to aberrant activation of resistance-associated gene programs [13]. In addition, dysregulated expression of the histone methyltransferase EZH2, driven by aberrant deposition of H3K27me3, has been implicated in the repression of tumor suppressor genes and development of resistance to immunotherapy [14]. Taken together, these seminal findings established epigenetic modifications not as passive bystanders, but as active and reversible regulators of tumor adaptive evolution, thereby positioning them as central determinants of therapeutic resistance (Fig. 1).

Importantly, these discoveries have driven a fundamental paradigm shift in cancer biology, from a framework centered on static genomic alterations to one that conceptualizes tumors as dynamic and adaptive systems shaped by epigenetic plasticity [15]. Tumor cells are recognized as active participants that deploy complex epigenetic regulatory networks to rapidly and reversibly reprogram gene expression, thereby establishing adaptive survival states under therapeutic stress [6, 16]. This intrinsic plasticity underlies not only the rapid emergence of resistance, the persistence of tumor heterogeneity, and the adaptive evolution of cancer. Despite substantial progress, current knowledge remains fragmented [9, 17]. Most reviews have focused primarily on individual epigenetic mechanisms in specific therapeutic contexts. Moreover, there remains a substantial gap between the mechanisms uncovered in basic research and the development of effective combination therapies. This is particularly true for the rational integration of epigenetic drugs with traditional treatments and immunotherapies, where notable knowledge and translational barriers persist [18].

This review revisits and extends Waddington's seminal concept of the “epigenetic landscape,” which was originally developed to describe cellular fate determination [19, 20]. Over time, this concept has evolved into a highly dynamic and multiscale regulatory network in tumor biology, encompassing DNA and RNA modifications, histone post-translational modifications, chromatin-remodeling complexes, and non-coding RNA networks [21, 22]. The intricate interactions within this network endow tumor cells with remarkable epigenetic plasticity, enabling them to dynamically modulate and stabilize gene expression programs that support survival under therapeutic stress [9, 15, 17]. This review comprehensive discusses how this plastic landscape governs therapeutic resistance, focusing on the four interconnected molecular layers outlined above and the regulatory networks driven by their crosstalk to facilitate rapid phenotypic adaptation. Based on this foundation, we explored how epigenetic reprogramming sustains critical resistance phenotypes, including stem cell-like drug-tolerant states, immunosuppressive tumor microenvironments, compensatory survival signaling, and enhanced DNA repair mechanisms. Furthermore, we evaluated emerging therapeutic strategies, from conventional epigenomic drugs to innovative approaches, including targeted protein degradation (TPD), epigenetic editing, and rational combination therapies, aimed at targeting this plasticity landscape. This comprehensive framework offers both conceptual insights and a translational pathway for combating cancer therapy resistance.

## Molecular pillars of the epigenetic landscape

**Fig. 2:**
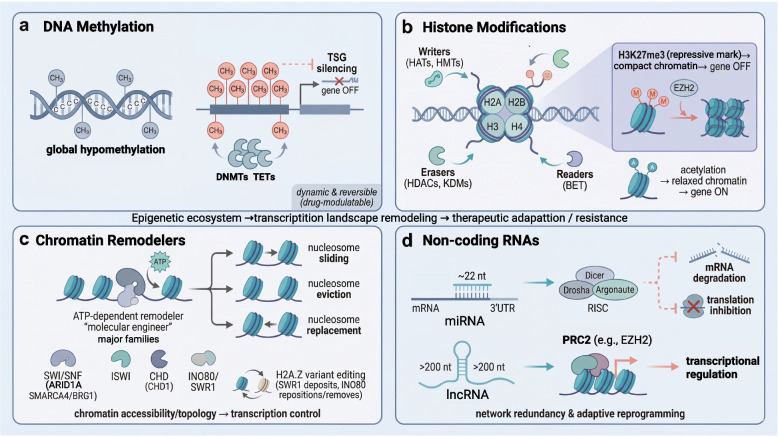
Molecular pillars of the epigenetic landscape and their roles in therapy resistance. **a** DNA methylation: Schematic representation of the cancer-specific pattern featuring global hypomethylation (associated with genomic instability) and focal promoter hypermethylation of CpG islands, resulting in transcriptional silencing of tumor suppressor genes (TSGs). **b** Histone modifications: Depiction of the reversible “histone code,” regulated by the balanced action of writers (e.g., HATs, HMTs), erasers (e.g., HDACs, KDMs), and readers (e.g., BET proteins). This model illustrates how specific marks (e.g., H3K27me3 for repression) dictate chromatin states and gene expression programs that are co-opted in cancer. **c** Chromatin-remodeling complexes (e.g., SWI/SNF and ISWI) hydrolyze ATP to slide, evict, or restructure nucleosomes, thereby regulating DNA accessibility and enabling dynamic transcriptional responses to therapeutic stress. **d** Non-coding RNAs: miRNAs regulate gene expression via mRNA degradation and translation inhibition, with PRC2 enhancing epigenetic regulation to reinforce resistance

The “epigenetic landscape” is a highly coordinated and dynamically responsive network composed of four molecular pillars: DNA methylation, histone modification, chromatin remodeling, and non-coding RNAs [23]. This system is hijacked by cancer cells and reshapes the transcriptional landscape to form the foundation for therapeutic resistance [23, 24]. This section provides an in-depth analysis of the core functions and regulatory logic of each pillar. Understanding their fundamental roles and how they operate synergistically is crucial for developing strategies to reverse resistance (Fig. 2).

### DNA Methylation

DNA methylation is a highly conserved and extensively studied core epigenetic modification that plays a pivotal role in maintaining the stability of gene expression patterns [25]. Its biochemical nature involves the addition of a methyl group to the cytosine of a CpG dinucleotide, resulting in the formation of 5-methylcytosine [26]. Genomic regions rich in CpG sequences, known as CpG islands, are often located in the promoter regions of genes and serve as critical sites for DNA methylation-mediated transcriptional regulation [27].

In normal cells, DNA methylation patterns function as essential epigenetic memory mechanisms that maintain cellular identity and genomic stability [25]. Typically, the promoter CpG islands of housekeeping genes remain unmethylated, ensuring basal transcriptional activity [28]. In contrast, tissue-specific genes, imprinted genes, and repetitive sequences in the genome undergo selective methylation. This intricate epigenetic regulation is crucial for preserving cellular identity, maintaining genomic stability, and preventing genetic rearrangements [29].

In cancer, this finely tuned regulatory system is profoundly disrupted and exhibits a characteristic pattern of global hypomethylation coupled with localized hypermethylation [30]. Global hypomethylation affects multiple regions of the genome, leading to genomic instability and the aberrant activation of oncogenes [31]. Conversely, localized hypermethylation specifically silences the promoters of numerous tumor suppressor genes, thereby inactivating their functions. Collectively, these epigenetic alterations drive tumorigenesis and therapeutic resistance [30, 32]. Notably, DNA methylation is not constant. Its dynamic and reversible nature can be modulated by demethylating agents, enabling therapeutic interventions that may promote cancer cell adaptation to stress and ultimately confer resistance [33, 34]. Therefore, DNA methylation is not merely a static imprint that maintains gene expression patterns but also a crucial regulatory element in tumor evolution, capable of dynamic reprogramming to endow tumor cells with phenotypic plasticity and adaptability [30, 33].

### Histone modifications

While DNA methylation provides a relatively stable epigenetic foundation, histone modifications represent a highly dynamic and reversible regulatory process. Post-translational modifications of histone tails, such as acetylation and methylation, together with their combinations, form a rich “histone code” that precisely regulates gene expression by directly altering chromatin structure or recruiting specific “reader” proteins [35, 36].

The dynamic equilibrium of histone modification is maintained by a finely tuned network of three functional proteins. “Writers” such as histone acetyltransferases (HATs) and methyltransferases (HMTs) add specific modifications and encode additional layers of epigenetic information [37]. “Erasers,” including histone deacetylases (HDACs) and demethylases (KDMs), remove these modifications, enabling signal erasure and resetting, and thus providing plasticity for epigenetic regulation [38]. “Readers” are proteins with bromodomains, chromatin-binding domains, and other modules that specifically recognize and bind to particular modifications (e.g., BET proteins recognize acetylated lysines). These proteins serve as bridges that link histone modifications to their downstream biological effects [39].

In normal cells, specific histone modification patterns precisely correspond to gene expression states [40]. Active gene promoters are enriched with activation marks such as H3K27ac, which loosen chromatin structure [41], while repressive regions are covered by marks such as H3K27me3 to maintain a heterochromatic state [42, 43]. However, this regulatory program is disrupted systematically in cancer. Aberrant activation of “writers” or overexpression of “erasers” leads to an imbalance in the global modification landscape [44, 45]. The consequences include erroneous silencing of tumor suppressor genes and aberrant activation of oncogenes and genes associated with cellular plasticity [44]. This large-scale dysregulation of histone modifications directly drives plasticity, uncontrolled proliferative potential, and therapeutic resistance of cancer cells, forming a critical epigenetic foundation for drug resistance [46].

### Chromatin-remodeling complexes

While DNA methylation and histone modifications are chemical marks of chromatin, ATP-dependent chromatin-remodeling complexes utilize the energy from ATP hydrolysis to physically move, reorganize, or replace nucleosomes. This dynamic process regulates chromatin accessibility and influences core biological processes such as transcription, DNA repair, and replication [47]. Such direct structural remodeling represents a crucial step in converting epigenetic information into functional outcomes [48].

Based on the structural characteristics of their core ATPase subunits, chromatin-remodeling complexes are classified into four main families: SWI/SNF, ISWI, CHD, and INO80/SWR1, each with distinct functional modes [49]. The SWI/SNF complex, the most extensively studied and frequently mutated complex in cancer, typically opens chromatin regions by sliding or evicting nucleosomes, thereby promoting transcriptional activation. Inactivating mutations in core subunits (e.g., ARID1A and SMARCA4/BRG1) can lead to the silencing of tumor suppressor pathways [50]. The ISWI family primarily regulates nucleosome spacing and chromatin assembly, often forming compact structures that exert transcriptional repression across the genome [51]. The CHD family (e.g., CHD1) drives directed nucleosome sliding, precisely controlling nucleosome occupancy at critical regions, such as promoters, thereby influencing gene expression [52]. The INO80 and SWR1 families focus on the regulation of the histone variant H2A.Z; SWR1 inserts it near promoters, whereas INO80 can remove or reposition it, functioning cooperatively to finely modulate local chromatin states and transcriptional activity [53].

Dysfunction of these remodeling complexes directly modifies the chromatin accessibility of regions harboring oncogenes or tumor suppressor genes, profoundly affecting the biological behavior and therapeutic response of tumor cells [54]. For instance, the loss of SWI/SNF function can lead to the physical silencing of key tumor suppressor pathways, thereby enhancing therapeutic resistance [55]. Conversely, aberrant remodeling activity may activate pro-survival or metastatic programs, driving adaptive resistance [56, 57]. Therefore, chromatin-remodeling complexes play a critical role in reshaping the transcriptional landscape of cancer cells to evade therapy through the direct regulation of genomic accessibility [58].

### Non-coding RNAs

Non-coding RNAs are functional molecules transcribed from non-coding regions of the genome that play a highly dynamic role in epigenetic regulation [59]. Based on their length, they are primarily classified as short non-coding RNAs (e.g., microRNAs) or long non-coding RNAs (lncRNAs) [60]. These molecules can precisely bind to DNA, RNA, or proteins and employ diverse mechanisms to finely regulate gene expression at the transcriptional, post-transcriptional, and epigenetic levels, thereby forming a complex, multilayered network [61].

MicroRNAs are single-stranded RNAs of approximately 22 nucleotides in length. They regulate gene expression post-transcriptionally by binding to the 3' untranslated region (UTR) of target mRNAs, guiding the RISC complex to mediate mRNA degradation or translational repression [62, 63]. LncRNAs, typically exceeding 200 nucleotides in length, exhibit diverse functional mechanisms. They can act as decoy molecules, competitively binding to microRNAs or transcription factors to regulate downstream pathways [64, 65]; serve as guide molecules, directing chromatin modification complexes (such as PRC2) to specific genomic loci to mediate local epigenetic remodeling [66, 67]; or function as scaffold molecules, assembling and stabilizing multifunctional protein complexes that coordinate transcriptional regulation or chromatin-remodeling processes [68, 69].

In cancer, dysregulation of the ncRNA network is a major driver of systemic drug resistance [70, 71]. MicroRNAs can rapidly establish multidrug resistance barriers by simultaneously repressing multiple apoptosis- or drug transport-related mRNAs [72]. LncRNAs can recruit epigenetic modification complexes (such as PRC2) to silence tumor suppressor genes, or act as scaffolds to activate pro-survival pathways (e.g., NF-κB/STAT3), facilitating dynamic reprogramming of resistance mechanisms [73, 74]. The interactive regulatory networks formed by these molecules enhance the adaptability and plasticity of tumor cells, providing a strong theoretical basis for their potential as therapeutic targets [65, 75].

This section systematically outlines the four molecular pillars constituting the epigenetic landscape. Although each mechanism is distinct, they are intricately interconnected, forming a highly dynamic and interdependent regulatory network [4] (Table 1). Under therapeutic stress, cancer cells hijack and remodel this network to synergistically suppress tumor suppressor pathways and activate pro-survival programs, thereby establishing a robust drug-resistant phenotype [76]. Understanding the cooperative interactions among these pillars is fundamental to unveiling the mechanisms of resistance and developing targeted therapeutic strategies. The following section examines how these molecular mechanisms are integrated to drive the complex biological processes of resistance.

**Table 1:** Functional comparison of epigenetic molecular pillars

Molecular cornerstone	Type	Main functions	Key enzymes	Mechanism	References
DNA Methylation	-	Global hypomethylation and local hypermethylation in cancer	DNMTs	Increased methylation of the promoter CpG island can silence key tumor suppressor genes, and its reversible process plays a core regulatory role in drug resistance adaptation	[77]
Histone Modifications	Writers	Dynamically regulates chromatin structure and gene expression, and leads to an imbalance in genome-wide modifications in cancer	HATs, HMTs;	Abnormal modification silences tumor suppressor genes and activates oncogenic/plasticity genes; drives cellular identity plasticity and therapeutic adaptation	[78]
	Erasers		HDACs, KDMs;		
	Readers		BET protein		
Chromatin-Remodeling Complexes	SWI/SNF	ATP hydrolysis energy is used to physically move, reorganize, or replace nucleosomes to directly regulate chromatin accessibility and topology	SMARCA4/BRG1, ARID1A	Abnormal function leads to the reduction of tumor suppressor gene accessibility and silencing; aberrant activation can drive pro-survival gene expression and promote drug resistance	[47]
	ISWI		SMARCA5/SNF2H		
	CHD		CHD1		
	INO80/SWR1		INO80, EP400		
Non-coding RNAs	miRNA	Post-transcriptional regulation, targeting multiple mRNAs to rapidly adjust cellular states	Dicer, Drosha, Argonaute	miRNA simultaneously inhibits multiple apoptosis- and drug transport-related genes to establish a multidrug resistance barrier	[79]
	lncRNA	As a decoy, guide, or scaffold molecule, it is precisely regulated at the transcriptional and epigenetic level	EZH2, SUZ12, EED	lncRNA recruit epigenetic complexes to silence tumor suppressor genes or activate pro-survival pathways	[80]

## Epigenetically driven molecular mechanisms of drug resistance

**Fig. 3:**
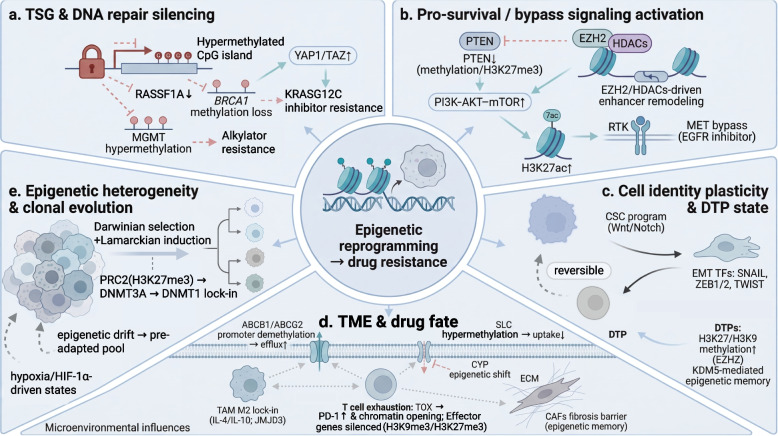
Epigenetic reprogramming and its role in cancer therapy resistance. **a** TSG and DNA repair silencing: Promoter hypermethylation of genes such as RASSF1A and BRCA1 inactivates key regulatory nodes, while MGMT hypermethylation directly confers resistance to alkylating agents. **b** Epigenetic downregulation of PTEN and EZH2-driven enhancer remodeling hyperactivates the PI3K–AKT–mTOR axis and facilitates bypass pathways (e.g., MET), sustaining survival signals under targeted therapy. **c** Cell identity plasticity and DTP state: Epigenetic modifiers (e.g., EZH2, KDM5) establish and maintain a drug-tolerant persister (DTP) state, reinforce cancer stem cell (CSC) programs, and facilitate epithelial-mesenchymal transition (EMT) through regulators like SNAIL and ZEB1. **d** Tumor microenvironment and drug fate: Epigenetic alterations in immune cells drive T-cell exhaustion (via TOX/PD-1) and M2-like tumor-associated macrophage (TAM) polarization. Cancer-associated fibroblasts (CAFs) are epigenetically locked into a pro-fibrotic state, while upregulated drug efflux pumps (e.g., ABCB1) reduce intracellular drug accumulation. **e** Epigenetic heterogeneity and clonal evolution: Pre-existing epigenetic heterogeneity and therapy-induced reprogramming interact through Darwinian selection and Lamarckian induction, leading to the expansion of resistant clones

Resistance to cancer treatment is a multilayered adaptive process, with dynamic remodeling of the epigenetic landscape serving as its core driving force [8]. Unlike genetic mutations, the reversibility and plasticity of epigenetic changes provide a unique molecular foundation for the rapid adaptation of tumor cells to therapeutic stress, while also creating critical therapeutic windows [4]. This section presents an integrated perspective of how the epigenetic landscape collaboratively regulates intrinsic gene programs, drives cellular identity shifts and population evolution, and remodels the tumor microenvironment, thereby collectively constructing a robust and flexible resistance network (Fig. 3).

### Silencing of tumor suppressor and DNA repair genes

By leveraging the dynamic reversibility of DNA methylation, cancer cells can reprogram the methylation landscape of specific genomic regions under therapeutic pressure. One of the most direct mechanisms driving drug resistance is the specific hypermethylation of two critical functional gene categories: tumor suppressor genes and DNA repair genes [81]. However, the silencing of these genes plays distinct roles in driving resistance; the former primarily promotes resistance to targeted therapies [9], whereas the latter drives resistance to chemotherapy by compromising genomic integrity, inducing mutations, and facilitating adaptive adaptation [82, 83].

Tumor suppressor genes monitor survival and proliferation signals by executing cell cycle arrest, senescence, or apoptosis programs; their epigenetic inactivation eliminates this surveillance. For example, RASSF1A, a key node linking the Hippo and RAS signaling pathways, frequently undergoes promoter hypermethylation in renal cell carcinoma and lung cancer [84, 85]. Silencing of RASSF1A disrupts Hippo pathway–mediated apoptotic control of RAS signaling, leading to hyperactivation of downstream survival pathways, such as AKT [85, 86]. Simultaneously, dysregulation of the Hippo pathway results in sustained activation of the transcriptional coactivator YAP1/TAZ, remodeling transcriptional programs to promote cell survival and proliferation [87, 88]. This epigenetic silencing of a critical signaling hub ultimately drives resistance to targeted RAS inhibitors by sustaining YAP1 activation [88]. In KRAS-mutant tumors, YAP1 activation mediated by RASSF1A deficiency has been identified as the core mechanism through which cancer cells evade direct KRASG12C inhibitor‒induced killing and develop acquired resistance [88, 89]. This clearly illustrates how the epigenetic shutdown of key tumor suppressor signals can systematically reprogram cellular states to circumvent targeted therapies.

Unlike tumor suppressor genes, the silencing of DNA repair genes directly affects genomic maintenance, resulting in dual and dynamic resistance effects. It can create temporary therapeutic vulnerabilities through “synthetic lethality” [90, 91], while also increasing genomic instability by impairing DNA repair, thereby providing a mutational foundation for subsequent resistance evolution [92, 93]. For example, hypermethylation of the BRCA1 promoter is a major mechanism underlying homologous recombination (HR) repair deficiency, and frequently occurs in triple-negative breast cancer [94]. This epigenetic silencing renders tumors initially sensitive to poly ADP ribose polymerase (PARP) inhibitors or platinum-based drugs; however, its reversibility poses a fundamental risk of resistance [95, 96]. Under therapeutic stress, loss of methylation can lead to re-expression of BRCA1, directly triggering acquired clinical resistance [95]. Key clinical trials, including ARIEL2, have confirmed that BRCA1 promoter methylation status serves as a biomarker for predicting PARP inhibitor efficacy, with dynamic changes directly correlating with reversal of treatment outcomes [97, 98]. Furthermore, the silencing of specific repair genes can directly result in drug failure at the biochemical level. One prime example is *MGMT*, which encodes an enzyme responsible for repairing DNA damage caused by alkylating agents. Hypermethylation of its promoter leads to the loss of this function and is a critical mechanism of tumor resistance to alkylating agents such as temozolomide [99]. In clinical studies of metastatic colorectal cancer, patients with *MGMT* promoter hypermethylation were reported to have a very low objective response rate (approximately 10%) to temozolomide monotherapy, establishing a clear causal relationship between this epigenetic change and drug resistance [99]. Accordingly, epigenetic silencing represents an adaptive process that is dynamically regulated by environmental pressure and can directly shape treatment outcomes.

### Activation of pro-survival and resistance signaling pathways

A core objective of cancer therapy is to block key signaling pathways that drive tumor survival and proliferation [100]. However, cancer cells can preprogram or dynamically rewire their signaling networks through epigenetic mechanisms, leading to treatment resistance [101, 102]. This process underscores the central role of epigenetic regulation in mediating adaptive responses in cancer cells [18].

#### Epigenetic hijacking of core signaling hubs

In tumor cell signaling networks, the PI3K-AKT-mTOR and RAS-RAF-MEK-ERK pathways are central to regulating cell growth, proliferation, and survival [103]. The development of resistance often arises from cancer cells directly reprogramming the regulatory architecture of these key pathways through epigenetic mechanisms, thereby restoring or maintaining pro-survival signaling outputs without relying on de novo mutations [1, 4].

Epigenetic hijacking of core signaling hubs is often achieved through dose-sensitive, finely tuned regulation [104]. For instance, hypermethylation of the phosphatase and tensin homolog (PTEN) gene promoter or the addition of H3K27me3 modifications progressively reduces its expression. While this subtle downregulation does not mimic complete PTEN loss, it is sufficient to push PI3K-AKT pathway activity beyond its functional threshold, thereby activating downstream survival programs and conferring resistance [105, 106]. The advantage of this regulatory mechanism lies in its plasticity, which allows cells to dynamically adjust signaling levels in response to environmental pressures, thereby enabling rapid adaptation [9]. Importantly, this hijacking process operates independently of mutations in pathway-driving genes [107]. Therefore, in targeted therapy, even if drugs successfully inhibit mutated kinases, cancer cells can compensate for the suppressed signaling flow through the aforementioned epigenetic reprogramming mechanisms or by restoring baseline signaling levels above the resistance threshold, leading to adaptive resistance [108, 109]. In several cases of early acquired resistance, no known resistance mutations have been detected; instead, global or focal chromatin remodeling has been observed [76, 110]. For example, in the HER2 signaling pathway, the tumor suppressor gene PPP2R2B is silenced by EZH2-mediated histone methylation at its promoter region, resulting in the repression of key signaling molecules, such as PP2A targets. Even in the absence of HER2 gene mutations or amplification, epigenetic silencing drives aberrant activation of the MAPK pathway or other downstream signaling pathways, leading to resistance to HER2-targeted therapy [111]. Epigenomic studies have demonstrated that, in MEK inhibitor–resistant cancer models, enhancer remodeling and changes in histone modifications (including elevated H3K27 acetylation) induce aberrant expression of key signaling proteins, thereby bypassing upstream inhibition and reactivating the entire ERK pathway. This process occurs independent of secondary mutations in BRAF or NRAS [110, 112]. These findings demonstrate that epigenetic mechanisms can reestablish pro-survival signaling outputs in the absence of new mutations, providing a flexible defense mechanism that operates independently of genotype.

#### Epigenetically mediated bypass activation and feedback loop reinforcement

In addition to epigenetically reprogramming core signaling hubs, tumor cells can adapt to therapeutic pressure through a broader process of adaptive network remodeling [113, 114]. At the core of this process is the ability of epigenetic mechanisms to rapidly and dynamically reshape the topology of the cellular signaling network, primarily through two interrelated strategies: bypass pathway activation and feedback loop reinforcement [115, 116].

In response to the targeted inhibition of key pathways (such as EGFR-RAS-ERK), tumor cells leverage their epigenetic plasticity to initiate adaptive responses. Epigenetic regulators such as EZH2 and HDACs are rapidly activated [117–119], driving the reprogramming of gene expression profiles. This ultimately results in the upregulation of bypass signaling pathways such as MET or the activation of alternative transcriptional programs, thereby sustaining tumor cell survival [117, 120, 121]. In lung cancer treated with EGFR inhibitors, therapeutic pressure can induce chromatin opening in specific enhancer regions, promoting MET expression through increased deposition of activation markers such as H3K27ac [119, 122, 123]. Elevated MET receptor expression then reinitiates downstream pro-survival signaling via a novel pathway (MET-GAB1-PI3K-AKT), effectively bypassing the inhibited EGFR pathway [119, 124]. This process highlights the rapidity and efficiency of epigenetically mediated adaptation, extending beyond the explanatory scope of classical Darwinian evolutionary models [119, 125].

Negative feedback loops are prevalent in cellular signaling networks and maintain signal homeostasis. However, these feedback mechanisms are often epigenetically hijacked [126]. When targeted drugs inhibit a pathway, they typically relieve the negative feedback inhibition of upstream or bypass signals, leading to a compensatory rebound of these signals [127, 128]. Initially, such rebound responses may be transient and unstable. Nevertheless, prolonged therapeutic pressure further modifies this response through epigenetic changes, thereby “locking” the transient rebound into a stable, persistent activation state [93, 108]. For example, inhibition of mTORC1 removes its negative feedback on upstream receptor tyrosine kinases (RTKs), resulting in a strong rebound in the PI3K-AKT signaling pathway [129]. With continued mTOR inhibition, epigenetic mechanisms can stabilize this rebound [1, 130].

More importantly, bypass activation and feedback reinforcement mutually promote each other. RTKs activated by bypass mechanisms such as MET can be part of a negative feedback loop [131]. Their dysregulated expression not only provides a direct escape route but also alters the feedback dynamics of the entire signaling network, further reducing sensitivity to the initial drug [132, 133]. In summary, epigenetic mechanisms, through both the vertical regulation of key hubs and the horizontal remodeling of network structures, establish a powerful and flexible defense system that drives rapid non-genetic adaptive resistance.

### Epigenetic reprogramming of cellular identity toward stem cell-like properties

In the dynamic interplay between tumor evolution and treatment response, cancer cells are not static entities but exhibit substantial plasticity, enabling them to adapt to microenvironmental and therapeutic pressures while maintaining a relatively stable genetic background [134]. Epigenetic regulation serves as the core engine driving this process, reprogramming gene expression networks to endow tumor cells with stem cell-like properties, lineage plasticity, and reversible drug-tolerant “dormant” states [9, 135]. These epigenetically driven identity shifts are key mechanisms in the development and maintenance of resistance and offer new perspectives for understanding and overcoming treatment failure [136].

#### Epigenetic regulation of Cancer Stem Cells (CSCs)

CSCs, a small subset of cells within tumors with self-renewal and multi-lineage differentiation potential, are considered to underlie tumor initiation, metastasis, relapse, and resistance. Differentiated tumor cells can acquire CSC-like properties through epigenetic reprogramming, whereas CSCs depend on sustained epigenetic regulation to maintain these properties [137].

Maintenance of CSCs typically involves the aberrant activation of embryonic development signaling pathways, such as Wnt/β-catenin and Notch, with epigenetic mechanisms playing a crucial regulatory role in this process [138, 139]. In acute myeloid leukemia (AML), loss-of-function mutations in DNMTs (e.g., DNMT3A) or TET enzymes disrupt normal hematopoietic differentiation programs, promoting the generation and maintenance of leukemia stem cells [140, 141]. In addition, histone modifications are implicated, with the histone methyltransferase EZH2 catalyzing H3K27me3 modification to suppress the expression of differentiation-related genes (e.g., Sox9), thereby enhancing the activation of stemness-associated signaling pathways (e.g., Wnt/β-catenin) and sustaining the stem cell state [142–144]. The core transcription factor network governing CSC stemness is regulated by epigenetic mechanisms [145]. In glioblastomas, inactivation of the histone demethylase KDM6A leads to elevated H3K27me3 levels, inhibiting genes that promote differentiation, thereby maintaining the CSC state [146, 147]. Reversing these aberrant modifications, for example, by using DNMT or EZH2 inhibitors, can induce differentiation and reduce stemness, further confirming the pivotal role of epigenetics in sustaining the CSC identity [140, 148].

#### Epigenetic regulation of lineage plasticity and Epithelial-Mesenchymal Transition (EMT)

Lineage plasticity refers to the ability of tumor cells to switch identities between different cell lineages, a critical strategy by which tumors adapt to therapeutic pressures. EMT is the most representative example of lineage plasticity, endowing cancer cells with migratory, invasive, and stem cell-like properties, and is closely associated with therapeutic resistance [138].

EMT initiation depends on core transcription factors, such as SNAIL, ZEB1/2, and TWIST, which are typically maintained in a repressed chromatin state under basal conditions [149–151]. In tumors, specific signaling pathways are activated in response to microenvironmental signals such as transforming growth factor (TGF)-β to induce the expression and function of these EMT transcription factors [152, 153]. For example, the histone demethylase KDM1A/LSD1 removes methylation marks from H3K4me2/1, repressing epithelial markers and promoting mesenchymal gene expression [154].

At key nodes of lineage transition, promoter regions of critical genes may exhibit a “bivalent” chromatin state, characterized by the coexistence of activating marks (H3K4me3) and repressive marks (H3K27me3) [92, 155]. This epigenetic configuration places genes in a highly plastic regulatory state, enabling cells to respond rapidly to external signals and to facilitate dynamic switching between different lineages [156, 157]. For example, under targeted therapy, lung adenocarcinoma cells can undergo epigenetic reprogramming to activate latent neuroendocrine lineage genes, transitioning into a drug-resistant small-cell lung cancer-like phenotype. This process involves the resolution of bivalent domains and activation of key lineage genes [156], a mechanism that has been widely observed across diverse cancer types [158, 159].

#### Drug-Tolerant Persister (DTP) cells: epigenetically mediated reversible drug resistance

Under initial therapeutic pressure, while the majority of sensitive tumor cells are eliminated, a small subset survives, entering a state of slow proliferation and metabolic reprogramming known as DTP cells [160]. DTPs do not arise from stable genetic mutations, and their resistance is temporary and reversible, being primarily driven by changes in the epigenetic landscape. Upon drug withdrawal, these cells resume proliferation and eventually develop more stable resistance [161].

DTP formation is reportedly accompanied by genome-wide chromatin remodeling [162]. For instance, during chemotherapy or targeted therapy, surviving DTPs exhibit epigenetic changes characterized by increased methylation of histone H3 at lysine sites (such as H3K27 and H3K9) and dynamic remodeling of the open chromatin architecture to maintain plasticity [162, 163]. This state can even establish epigenetic memory. Even after drug withdrawal, treatment-induced chromatin landscape alterations (e.g., reduced H3K27me3 at key gene promoters) may persist, enabling cells to more rapidly initiate resistance mechanisms upon re-exposure to the same treatment [162, 163]. Targeting enzymes that mediate these changes, such as EZH2 and the KDM5 family, effectively clears DTPs and delays the onset of resistance [164–166]. Plasticity of cellular identity represents the core dimension of epigenetically driven drug resistance. By inducing stemness, driving lineage conversion, or establishing reversible drug-tolerant states, epigenetic reprogramming provides tumor cells with multidimensional, dynamic escape strategies.

### Epigenetic regulation of the tumor microenvironment and drug disposition interfaces

Tumor resistance arises not only from genetic and epigenetic variations within tumor cells, but also from dynamic interactions between tumor cells and the microenvironment, as well as from the regulation of drug transport and metabolic processes [21]. Epigenetic mechanisms play a central role in this process, directly regulating the expression of drug disposition–related genes and remodeling the tumor microenvironment. Thus, epigenetic regulation promotes resistance through multiple mechanisms, including intrinsic cellular mechanisms and external ecological factors [9, 23].

#### Intrinsic biochemical barriers: epigenetic reprogramming of drug disposition–related genes

Within cancer cells, epigenetic mechanisms reprogram drug disposition–related genes to reduce intracellular concentrations of effective drugs [167, 168]. The efficacy of a drug primarily depends on its intracellular concentration, a process largely regulated by the ATP-binding cassette (ABC) and solute carrier (SLC) transporter families, whose expression levels are tightly controlled by epigenetic mechanisms [169, 170]. The upregulation of drug efflux pumps (e.g., ABCB1/P-gp and ABCG2/BCRP) is a key factor in multidrug resistance (MDR) [171]. Studies have shown that this upregulation is not always due to gene amplification but is often associated with epigenetic derepression mechanisms. For example, DNA demethylation in the promoter regions of ABCB1 and ABCG2 relieves transcriptional repression, leading to overexpression [171, 172]. In contrast, the SLC transporter family, which mediates intracellular drug uptake, is often silenced. For instance, downregulation of copper or organic cation transporters, which are responsible for the uptake of platinum-based drugs, directly results in insufficient drug uptake and resistance [173, 174]. This silencing of expression is often closely linked to promoter hypermethylation and the accumulation of repressive histone modifications (e.g., H3K27me3 or H3K9me3), which prevent the opening of these transport entry points and reduce drug uptake at the source [167, 175].

After cellular entry, drugs must also undergo metabolism to exert their effects or be eliminated. The cytochrome P450 (CYP) enzyme family is primarily responsible for drug metabolism [176, 177]. Epigenetic regulation via DNA methylation or histone modifications in the regulatory regions of specific CYP genes can repress or upregulate CYP expression, leading to dysregulated drug metabolism [178–180]. A key feature of this intrinsic biochemical barrier is its reversible and dynamic nature, which allows cancer cells to flexibly adjust the expression of relevant genes in response to the presence and intensity of therapeutic pressure, thereby achieving an optimal balance between survival and proliferation [4].

#### External ecological remodeling: epigenetic regulation of the immune and stromal microenvironment

Cancer cells not only remodel themselves but also actively reshape the surrounding microenvironment, establishing a protective ecological niche. By secreting various signaling molecules, cancer cells continuously regulate the functional state of immune and stromal cells within the tumor microenvironment. These changes are often mediated and sustained by persistent epigenetic reprogramming [181].

An actively functioning immune system is critical for eliminating cancer cells. Consequently, cancer cells have evolved strategies to systematically suppress antitumor immunity via epigenetic mechanisms. Cytokines secreted by cancer cells (e.g., interleukin [IL]−4 and IL-10) can induce macrophage polarization toward the pro-tumor M2 phenotype [182, 183]. Promoters and enhancers of M2 polarization-associated genes have been reported to undergo characteristic histone modifications, such as activation of the H3K27 demethylase JMJD3 and other modifications (e.g., histone acetylation) [184, 185]. Changes in DNMT activity also contribute to this process, enabling tumor-associated macrophages (TAMs) to stably maintain their pro-tumor functions and to establish a robust immune-suppressive barrier [186]. In the tumor microenvironment, sustained antigen stimulation leads to T-cell exhaustion, a process accompanied by profound epigenetic remodeling [187]. The transcription factor TOX is a key driver of this reprogramming; it promotes chromatin accessibility of exhaustion-related inhibitory receptors (e.g., programmed death-1 [PD-1]) while depositing repressive marks, such as H3K9me3 and DNA hypermethylation, thereby keeping effector function genes in a state of long-term silencing [188, 189]. These stable epigenetic changes represent a major obstacle to functional recovery, causing T cells to remain dysfunctional even after immune checkpoint inhibitor therapy, thereby establishing a critical mechanism of tumor immune escape [190, 191].

In addition to immune suppression, a dense extracellular matrix (ECM) and fibrosis act as physical barriers that impede effective infiltration of chemotherapy drugs and immune cells into tumor cores [192]. Cancer cells induce epigenetic reprogramming of cancer-associated fibroblasts (CAFs), transforming them into a persistently activated state that secretes large amounts of ECM, thereby forming a physical barrier that obstructs drug and immune-cell infiltration. This activated state is further sustained by epigenetic memory, which markedly limits treatment effectiveness [193–195]. Once established, CAFs autonomously maintain their profibrotic phenotype through epigenetic memory, thereby continuously reinforcing this physical barrier.

### Epigenetic heterogeneity within tumors and treatment-driven clonal evolution

Epigenetic heterogeneity within tumors is a major driver of treatment failure [196], with plasticity far exceeding that of genomic mutations, making it a key force in adaptive evolution [6]. Understanding its origins and evolutionary trajectories under therapeutic pressure is critical.

#### Origins and dimensions of epigenetic heterogeneity

Before any therapeutic intervention, solid tumors are not homogeneous cell populations but rather complex ecosystems composed of cells with diverse epigenetic states [6]. This inherent heterogeneity provides a potential foundation for adaptive changes, creating conditions that allow tumors to select advantageous clones under therapeutic pressure [197]. During continuous cell proliferation, replication of DNA methylation patterns and histone modifications is accompanied by a certain frequency of random errors, a phenomenon known as epigenetic drift [198]. These random, non-directed epigenetic changes induce considerable diversity within cell populations. Even in genetically identical cancer cell populations, this process leads to the spontaneous emergence of subpopulations with distinct epigenetic profiles, forming potential seeds for resistance [199–201].

Many tumors are thought to follow a hierarchical organizational model, with CSCs at the top, possessing self-renewal and multi-lineage differentiation potential [138]. CSCs and their differentiated progenies (progenitor cells and terminally differentiated cells) inherently exist in different epigenetic states that align with their developmental lineage positions and functional roles [202]. CSCs typically exhibit highly plastic epigenetic states, enabling them to respond flexibly to external signals by entering dormancy or initiating differentiation [203]. This intrinsic developmental epigenetic heterogeneity naturally leads to differences in treatment sensitivity among tumor cell populations. For example, quiescent CSCs are inherently resistant to chemotherapy drugs that target proliferation, and their epigenetic plasticity makes them prone to acquiring drug resistance under therapeutic pressure [204, 205].

The tumor microenvironment is highly spatially heterogeneous, with gradients in oxygen, nutrients, pH, and growth factor concentrations [206]. These microenvironmental signals instruct tumor cells to undergo adaptive epigenetic reprogramming. For example, hypoxia in the tumor core activates transcription factors such as HIF-1α, thereby remodeling the local chromatin environment and driving cells into a more invasive and drug-resistant state [207]. Likewise, physical contact with stromal cells (e.g., fibroblasts) or paracrine signaling can induce epigenetic changes in cancer cells, resulting in notably distinct epigenetic profiles across various tumor regions [208–210]. Collectively, random drift, developmental hierarchy, and microenvironmental cues shape a multidimensional dynamic pool of epigenetic heterogeneity, suggesting that subpopulations with potentially drug-resistant epigenetic traits may already exist at the onset of treatment.

#### Therapy-driven selection and induction of clonal evolution

Cancer therapy serves as a critical selective pressure that drives tumor progression. It not only selects pre-existing subpopulations of cells but also triggers widespread epigenetic reprogramming in response to this pressure, thereby driving the evolution of epigenetic heterogeneity [200, 211]. These therapy-driven effects are primarily realized through two core mechanisms.

Darwinian selection is a classic evolutionary model. When drugs are applied to tumors, the majority of cells sensitive to epigenetic changes are rapidly eliminated [212], while those pre-equipped with drug-resistant traits, through mechanisms such as epigenetic drift, survive and expand [213, 214]. This pre-existing epigenetic heterogeneity provides the foundation for the development of resistance. For instance, in ALK-positive lung cancer exposed to crizotinib, pre-existing resistant subclones can maintain survival signals through epigenetic reprogramming, allowing them to expand exponentially and eventually dominate the population [8, 9, 215]. Recent studies using algorithms such as epiCHAOS have further confirmed that tumors with high single-cell epigenetic heterogeneity scores exhibit poor responses to treatment, providing evidence that pre-existing resistant subclones directly drive therapeutic failure [6, 210].

In addition to passive selection, therapeutic pressure serves as a powerful biological signal that actively triggers rapid and dynamic epigenetic adaptive reprogramming in cancer cells [160]. This mode is akin to Lamarckian inheritance. In response to drug exposure, many initially sensitive cells do not immediately die but enter the DTP state described earlier [216]. For example, EGFR inhibitors can drive cells into a dormant DTP state for temporary survival by upregulating EZH2 and catalyzing H3K27me3 deposition, among other epigenetic mechanisms [217–219]. Therapy-induced reprogramming provides a critical window for the subsequent evolution of more stable resistance mechanisms [220]. Thus, therapy efficiently drives the evolution of resistant cell populations through the synergistic effects of Darwinian selection and Lamarckian induction.

#### From plastic adaptation to the stabilization of drug resistance

Under therapeutic pressure, tumor populations evolve from dynamic plastic adaptations to stable, fixed states, ultimately resulting in the formation of a genetically heterogeneous population dominated by resistant clones with stabilized epigenetic states [57, 221].

Whether originating from pre-existing resistant subclones or from cells recovering into DTPs, these cells serve as progenitors for the clonal expansion of drug resistance [160]. During treatment or treatment intervals, these clones gradually replace the original sensitive cell population through mechanisms such as sustained gene expression regulation and enhancer remodeling, ultimately dominating the tumor at relapse [8, 222]. Single-cell multi-omics tracking studies have confirmed that the cellular composition of relapsed tumors undergoes fundamental changes compared with pretreatment tumors and is typically dominated by clones that gain selective advantages under therapeutic pressure [222]. The evolution of clonal architecture is a direct manifestation of acquired clinical resistance.

To maintain and stably transmit drug resistance, initially reversible adaptive states, such as the DTP state, which rely on dynamic mechanisms, such as histone modifications, must be converted into a more stable, heritable epigenetic memory. In this process, DNA methylation plays a critical role in stabilizing resistance [92]. The core mechanism involves the cooperation of various epigenetic modifiers; for example, while the H3K27me3 modification deposited by the PRC2 complex can silence genes, its fidelity during cell division is limited [163, 223]. For example, DNMT3A can be recruited to target gene regions of PRC2, where it initiates de novo DNA methylation [224]. Subsequently, the maintenance methyltransferase DNMT1 accurately replicates this methylation pattern, establishing a long-term stable gene-silencing state [225]. Through this cascade mechanism—in which histone modifications guide and DNA methylation executes and stabilizes—tumor cells can convert pro-survival gene expression patterns (e.g., silencing of tumor suppressor genes) established under therapeutic pressure into a stable, drug-resistant epigenetic landscape [226].

The outcome of tumor evolution is the formation of a cell population whose clonal structure and epigenetic features have been profoundly reshaped. This population is dominated by a small number of drug-resistant clones that have emerged through selection and reprogramming under therapeutic pressure. Key resistance–related epigenetic alterations (e.g., aberrant methylation of specific genes) are stably locked, establishing a clinically persistent drug-resistant phenotype [227]. Understanding the complete evolutionary path, from heterogeneity to stabilization of resistance, provides a theoretical foundation for developing intervention strategies and blocking the formation of resistance.

## Reversal strategies targeting the epigenetic landscape

The dynamic reversibility of epigenetic regulation offers a valuable therapeutic window for overcoming treatment resistance. Interventions that directly target these mechanisms, known as epigenetic drugs, present unprecedented opportunities to restore tumor sensitivity to existing therapies [228]. This section systematically reviews strategies ranging from classical epigenetic modifier inhibitors to novel approaches targeting reader proteins, as well as multimodal combination therapies. We explore how these interventions can reverse resistance mechanisms at the molecular level and examine the challenges they face in clinical translation in order to provide a theoretical framework and forward-looking perspective for developing more intelligent and precise anti-resistance strategies.

### Classical strategies targeting epigenetic modifying enzymes

Epigenetic modifying enzymes, acting as “writers” and “erasers” of gene expression regulation, are critical targets in the development of epigenetic drugs [229]. By inhibiting the activity of these key enzymes, the epigenetic landscape of cells can be globally or selectively reshaped, thereby reversing drug-resistant phenotypes driven by aberrant epigenetic states [230].

#### DNMT Inhibitors (DNMTi) : demethylation and resensitization

DNA methylation, as a “writer” catalyzing the methylation of DNA cytosine, plays a pivotal role in gene silencing, particularly through the hypermethylation of tumor suppressor gene (TSG) promoters, which is a key event in tumorigenesis [33, 231]. DNMTi, among the earliest epigenetic drugs to enter clinical use, reverse the epigenetic silencing of key genes, thereby restoring normal regulatory pathways and resensitizing drug-resistant cells [231].

First-generation DNMTi, such as azacytidine and decitabine, are nucleoside analogs. During cell proliferation, they are incorporated into newly synthesized DNA strands, where they become irreversibly trapped and deplete maintenance methyltransferases [33, 232]. This results in passive genome-wide demethylation, which reactivates TSGs silenced by hypermethylation [32, 233]. In ovarian cancer models, decitabine has been shown to reverse platinum resistance by remodeling DNA repair pathways [234]. [23, 230]Notably, the effects of DNMTi extend beyond the restoration of individual gene functions. They can systematically reshape the entire cellular signaling network. For example, they induce the demethylation of endogenous retroviral elements (ERVs), activating a viral mimicry state: the double-stranded RNA produced by ERV transcription is recognized by intracellular pattern recognition receptors, leading to enhanced tumor immunogenicity through activation of the MAVS pathway and increased type I interferon production [23, 230]. These drugs are widely used in the treatment of hematologic malignancies such as myelodysplastic syndromes and AML [235]. In solid tumors, although monotherapy activity is limited, their potential as resensitizing agents in combination with conventional chemotherapy or targeted therapies is being extensively explored [236]. Preclinical studies have confirmed that pretreatment with low-dose DNMTi can effectively reverse platinum resistance in solid tumors, including ovarian cancer and triple-negative breast cancer [233, 234, 236].

#### HDAC Inhibitors (HDACi) : acetylation control and synergistic combination strategies

HDACs are key epigenetic regulators of chromatin structure and gene transcription [237]. In drug-resistant tumors, overactive HDACs often downregulate crucial genes involved in apoptosis, cell cycle regulation, and drug sensitivity [238, 239]. HDACi inhibit HDAC activity, increase histone acetylation, and loosen chromatin structure, which in turn promotes the re-expression of silenced genes [240].

HDACi are multifunctional agents that not only regulate histone acetylation but also modulate the acetylation of numerous non-histone substrates, such as HSP90, α-tubulin, and p53. These modifications broadly affect multiple cellular processes, including protein stability, cytoskeletal dynamics, and signal transduction [241]. Although monotherapy with HDACi has been associated with limited efficacy in solid tumors, their combination with chemotherapy, targeted therapy, or radiotherapy frequently yields synergistic effects. For instance, in non-small cell lung cancer, HDACi (e.g., quisinostat) can reverse resistance driven by EMT, enhancing sensitivity to ALK inhibitors (e.g., crizotinib) [242]. Furthermore, HDACi can reverse resistance to MEK inhibitors (e.g., trametinib) by altering enhancer reprogramming and upregulating MAPK negative regulators, thereby achieving a synergistic antitumor effect [112]. Although early pan-HDACi, such as vorinostat and romidepsin, were approved for clinical use, their toxicity has limited their widespread application [243]. Currently, research is focused on the development of highly selective inhibitors targeting specific HDAC subtypes, particularly HDAC6, which retain immunomodulatory activity while substantially reducing toxicity [243, 244]. HDAC6 inhibitors (e.g., ricolinostat) have shown better tolerance in preclinical models and can synergize with immune checkpoint inhibitors (ICIs) by modulating PD-1 ligand 1 (PD-L1) expression and T-cell function [245]. As of 2025, several clinical trials evaluating selective HDACi in combination with ICIs are underway [246].

#### HMT/KDM inhibitors: methylation reprogramming

Histone methylation is a more complex and specific modification, precisely regulated by HMTs and KDMs [247]. Different methylation sites and states (mono-, di-, or tri-methylation) are closely associated with gene activation or repression. Inhibitors targeting these key enzymes have the potential to achieve precise epigenetic reprogramming [248].

The core advantage of these inhibitors is their potential for precise reprogramming. Drugs targeting specific methyltransferases can more accurately intervene in epigenetic pathways associated with particular drug resistance phenotypes. For example, EZH2 inhibitors target EZH2, a key enzyme that catalyzes the transcriptionally repressive mark H3K27me3. EZH2 overactivation can silence TSGs and promote resistance [249, 250]. EZH2 inhibitors have demonstrated efficacy in various hematologic and solid tumors, with their mechanisms of reversing resistance primarily involving the reactivation of silenced target genes, alteration in cell differentiation states, and modulation of the tumor immune microenvironment [251, 252]. In AML, EZH2 inhibitors reduce H3K27me3 levels, open chromatin, and enhance the DNA damage response, thereby increasing sensitivity to chemotherapy [218]. Similarly, inhibitors targeting histone demethylases, such as LSD1, have shown potential to overcome resistance by modulating specific gene expression profiles. For example, in AML, LSD1 inhibitors currently in clinical trials can overcome resistance by reshaping the epigenome of CD8^+^ T cells and enhancing antitumor immunity [253, 254] (Table 2).

**Table 2:** Clinical trials of epigenetic drugs targeting tumor drug resistance (2020–2025) (clinicaltrials.gov)

Drug candidate/strategy	Type	Cancer type	Status	Phase	NCT number
BMS-986253	DNMTi	Myelodysplastic syndromes	Terminated	I/II	NCT05148234
Chidamide	HDACi	Non-Hodgkin's lymphoma	Recruiting	I/II	NCT05370547
JBI-802	HDACi	Solid tumors	Unknown status	I/II	NCT05268666
JBI-802	HDACi	Non-small cell lung cancer	Recruiting	II	NCT07207395
Seclidemstat	HDACi	Ewing sarcoma	Enrolling by invitation	I/II	NCT05266196
IMG-7289	HDACi	Essential thrombocythemia + polycythemia vera	Active, not recruiting	II	NCT04262141
CTS3497	HDACi	Solid tumors + lymphomas	Recruiting	I/II	NCT06971523
SHR2554	HDACi	Follicular lymphoma	Not yet recruiting	II	NCT06368167

#### Limitations of classic drugs and future directions

Although the aforementioned classic inhibitors have pioneered epigenetic therapy, their clinical applications have yet to overcome numerous challenges. First, classic DNMTi and pan-HDACi have insufficient selectivity and specificity [255], leading to genome-wide epigenetic disturbances and dose-limiting toxicity [256]. Second, their limited exposure to solid tumor tissues and modest monotherapy efficacy make their clinical value highly dependent on the development of combination therapies [257, 258]. Furthermore, tumor cells can acquire resistance through mechanisms such as epigenetic dysregulation, compensatory signaling pathway activation, and immune evasion, which undermine the long-term therapeutic effectiveness [259].

To address these limitations, the development of next-generation drugs is progressing along several key avenues, with the primary focus on creating highly selective inhibitors (e.g., targeting specific HDAC isoforms) to enhance the therapeutic window and reduce off-target effects [260]. In response to complex resistance networks, dual- or multitarget inhibitors (e.g., those that inhibit both HDAC and BET) are being explored to synergistically block resistance pathways [261, 262]. In terms of mechanisms, covalent inhibitors and allosteric modulators, through irreversible binding or regulation of allosteric sites, hold promise for achieving more durable and specific targeted inhibition [263]. Additionally, novel drug designs, such as platinum prodrugs that combine DNMTi and HDACi, aim to improve drug stability and bioavailability, a topic addressed in detail in later sections [264]. In summary, the iteration of classic epigenetic drugs drives a paradigm shift from broad-spectrum approaches to precise targeting [258].

### Targeting epigenetic readers: interfering with the interpretation of epigenetic information

The flow of epigenetic information follows a dynamic “writing–reading–erasing” cycle. Reader proteins, through their specific structural domains, recognize and bind to particular post-translational modifications on histones, interpreting these chemical marks as biological signals that regulate gene expression [265, 266]. In tumor cells, reader proteins are often hijacked to activate the transcription of key oncogenes or to sustain malignant phenotypes, thereby playing a critical role in the development and maintenance of resistance [267, 268]. Therefore, targeting reader proteins to block the interpretation of epigenetic signals is a promising strategy for directly disrupting oncogenic signaling and offers an attractive avenue for reversing resistance [269].

#### BET Inhibitors (BETi) : blocking acetylation-mediated signaling

Among multiple families of epigenetic readers, the BET protein family, particularly BRD4, has emerged as one of the most extensively studied and rapidly translated clinical targets [270, 271]. These proteins recognize histone acetylation marks and recruit transcriptional complexes to gene regulatory regions, thereby activating the transcription of target genes [272]. In several cancers, BET proteins are aberrantly enriched in super-enhancer regions, continuously activating key oncogenes and promoting the development of drug resistance [273].

BETi competitively bind to the bromodomain, displacing it from chromatin, thereby effectively suppressing the transcription of oncogenes driven by super-enhancers and inducing tumor cell apoptosis [161]. In reversing drug resistance, BETi have multifaceted potential. First, they suppress the expression of super-enhancer-dependent pro-survival genes (e.g., GPX2, ALDH3A1, MGST1), eliminate residual drug-resistant tumor cells (e.g., DTPs), and restore sensitivity to original therapies [161]. Second, BETi downregulate anti-apoptotic proteins, such as BCL-2, upregulate pro-apoptotic pathways, and enhance cell death induced by other therapeutic agents [274, 275]. Third, BET proteins regulate HR genes (e.g., RAD51, BRCA2). BETi weaken DNA repair capacity by inhibiting HR-related gene transcription, and when combined with PARP inhibitors, they induce synthetic lethality in HR-deficient tumors, substantially enhancing therapeutic efficacy [276].

Despite robust preclinical evidence, the efficacy of BETi as monotherapy for solid tumors remains limited, with adverse events potentially hindering treatment adherence. The rapid onset of acquired resistance is the primary barrier to their clinical application [277]. By 2025, numerous BETi had entered various phases of clinical trials, targeting both hematologic malignancies and a range of solid tumors; however, none have yet been approved for clinical use. The key to future development lies in identifying predictive biomarkers to accurately select the patient populations most likely to benefit from treatment [278, 279].

#### Targeting other reader proteins

As our understanding of epigenetic regulatory networks advances, the pivotal role of reader proteins beyond the BET family in tumor resistance is increasingly being recognized, thereby paving the way for novel drug development strategies.

The YEATS domain, recently identified as a reader module capable of recognizing histone acetylation and butyrylation, plays a pivotal role in epigenetic regulation [280]. ENL and AF9 are representative proteins containing the YEATS domain and are key drivers of various malignancies, including mixed-lineage leukemia–rearranged acute leukemia [267]. These proteins recruit transcriptional activation complexes to the promoters of specific genes such as HOXA9, thereby maintaining the malignant state of leukemia cells [267, 281]. Small-molecule inhibitors targeting the ENL YEATS domain have demonstrated potent antileukemic activity in preclinical models, effectively suppressing target gene expression and inducing cell differentiation and apoptosis [281, 282]. Mechanistically, ENL/AF9 functions partly in synergy with, but also distinctly from, BET proteins [281, 283]. Targeting ENL/AF9 offers a promising approach for treating patients with leukemia who have primary or acquired resistance to BETi, potentially in synergy with BET or DOT1L inhibitors [284]. However, owing to the high homology between ENL and AF9, the development of selective inhibitors remains challenging [265]. Currently, no such agents have entered clinical trials. Although preliminary studies have explored their potential role in solid tumors, the mechanisms underlying their ability to reverse resistance, as well as their overall efficacy, warrant further investigation [285, 286].

CHD4, the core ATPase subunit of the NuRD chromatin-remodeling complex, regulates chromatin accessibility and gene expression by recognizing histone methylation marks and hydrolyzing ATP to mobilize nucleosomes [287]. The role of CHD4 in DNA damage repair (DDR) is context-dependent. In the presence of BRCA2 mutations, loss of CHD4 stabilizes replication forks, resulting in resistance to PARP inhibitors and platinum-based chemotherapy [288]. Conversely, silencing of CHD4 increases DNA accessibility and induces spontaneous DNA damage, enhancing cellular sensitivity to DNA-damaging agents [289]. Given its complex functions and critical role in maintaining genomic stability, the development of safe and specific CHD4 inhibitors poses considerable challenges [287, 290]. Currently, small-molecule inhibitors that directly target CHD4 are in the early stages of development; however, genetic intervention studies, such as RNAi, have revealed that CHD4 inhibition promotes apoptosis, halts cell cycle progression, and downregulates MEK/ERK signaling, thereby enhancing the sensitivity of gastric cancer cells to cisplatin [291]. In ovarian cancer models, targeting CHD4-related pathways has shown promise in overcoming therapeutic resistance [292]. These findings underscore the therapeutic potential of targeting chromatin-remodeling complexes to overcome resistance.

Targeting epigenetic reader proteins provides a rich array of potential therapeutic targets for treating drug resistance [4]. BETi-based strategies have established the feasibility of this approach in preclinical and clinical studies but have also exposed challenges, such as modest single-agent activity and the emergence of secondary resistance [293]. Deeper insight into emerging reader proteins, such as ENL/AF9 and CHD4, together with the development of novel inhibitors, is expected to enable more precise and synergistic targeting approaches that can disrupt the epigenetic regulatory networks that sustain tumor resistance [294, 295].

### Next-generation intervention paradigms: epigenetic editing and targeted degradation

Classical epigenetic drugs have the potential to reverse drug resistance in clinical settings. However, their broad scope of action and substantial off-target effects have prompted researchers to explore more precise and efficient next-generation intervention paradigms [296]. Currently, emerging technologies such as precise epigenetic editing and TPD are fundamentally transforming how we regulate the epigenetic states of cancer cells, offering entirely new pathways to overcome treatment resistance [297].

#### Precision epigenetic editing

Precision epigenetic editing aims to achieve long-term regulation of key gene expression through precise, programmable epigenetic modifications at specific genomic loci without altering the DNA sequence itself [298]. This technology employs a catalytically inactive Cas9 variant (dCas9) as a programmable DNA-binding module. Targeted regulation of specific genomic sites can be achieved by fusing dCas9 with functional domains, such as transcriptional activators, repressors, or epigenetic modifying enzymes (e.g., methyltransferases or demethylases) [299]. Using a designed guide RNA (sgRNA), this fusion protein can be precisely directed to the promoters or enhancer regions of drug resistance–related genes, thereby reshaping the local epigenetic landscape [300].

Among the various gene-editing tools, the CRISPR/Cas9 system is particularly favored owing to its design flexibility and targeting precision. Its application enables programmable activation or suppression of specific gene expression programs, primarily through two strategies: CRISPR activation (CRISPRa) and CRISPR interference (CRISPRi/CRISPRoff). The CRISPRa system works by fusing dCas9 with a transcriptional activation module, enabling the specific reactivation of TSGs silenced by epigenetic modifications such as hypermethylation [301, 302]. This strategy can be used to upregulate the expression of apoptosis-related or drug-sensitive genes in resistant cells, thereby restoring their response to treatment [302]. Further studies have shown that activating endogenous immune-related genes in tumor cells via CRISPRa can enhance tumor immunogenicity, thereby triggering a robust antitumor immune response and offering a new approach for combination immunotherapy [303]. Both in vitro and in vivo experiments have confirmed that CRISPRA-mediated transcriptional activation of target genes significantly inhibits tumor growth [301, 303].

In contrast, CRISPRi and the more advanced CRISPRoff system achieve targeted, precise, and sustained gene silencing by fusing transcriptional repressors (e.g., the KRAB domain) or DNMTs [304]. This strategy can be employed to specifically downregulate the expression of key genes that drive drug resistance, thereby restoring tumor cell sensitivity to chemotherapy [305]. The CRISPRoff system establishes heritable DNA methylation modifications in the target gene region and maintains stable gene silencing across long-term cell passages [305]. This silencing state can be reversibly lifted by the corresponding CRISPRon system, enabling dynamic regulation of gene expression [306]. This precise, programmable gene expression intervention capability offers a promising approach to directly block the activation of resistance signaling pathways at the source [305].

#### TPD

TPD technologies employ a mechanism that is distinct from traditional inhibition strategies. The core principle involves inducing the degradation of disease-associated target proteins, rather than merely blocking their function [307]. This field primarily includes two tools: proteolysis-targeting chimeras (PROTACs) and molecular glues [308].

PROTACs are bifunctional molecules, with one end binding to the protein of interest (POI) and the other recruiting an E3 ubiquitin ligase. By forming a POI–PROTAC–E3 ternary complex, PROTACs direct the target protein for ubiquitination and subsequent proteasome-mediated degradation [309]. Compared with traditional inhibitors, PROTACs offer unique advantages: first, they can target traditionally undruggable proteins, such as scaffold proteins lacking catalytic active sites [309, 310]; second, they operate through a catalytic cycle, achieving profound depletion of the target protein at very low concentrations [309]; finally, by eliminating the target protein, PROTACs can abolish all of its functions and potentially overcome acquired resistance due to binding site mutations [309, 311]. In epigenetics, PROTACs have been used to degrade various key writer, reader, and eraser proteins [311]. For instance, PROTACs targeting BET family proteins (especially BRD4) have shown superior antitumor activity compared with corresponding inhibitors across multiple cancer models [312, 313]. Similarly, PROTACs targeting the "writer" EZH2 and the "eraser" HDACs have demonstrated potential in reversing resistance in preclinical studies [314, 315]. According to recent data, dozens of PROTAC molecules have entered clinical trials, including candidates targeting epigenetic proteins, highlighting the broad translational potential of this strategy [309].

Molecular glues are a class of small molecules that can induce or stabilize a novel protein–protein interaction interface, thereby “gluing” the target protein to an E3 ligase and facilitating its degradation [316]. Classic examples include thalidomide and its derivatives, which induce the binding and degradation of the transcription factor IKZF1/3 by interacting with the E3 ligase CRBN [316, 317]. Although the discovery of molecular glues has largely relied on serendipity and lacks the clear rational design principles available for PROTACs, their compact molecular structure and improved druggability make them an exceptionally attractive research direction [318]. The development of molecular glues targeting epigenetic regulatory proteins is a cutting-edge area in drug discovery [319].

#### Technical challenges and pathways to clinical translation

Despite the promising prospects of precision epigenetic editing and TPD technologies, the transition from laboratory to clinical applications faces three core challenges: efficient delivery, safety, and regulatory oversight.

Efficient and safe delivery remains the primary bottleneck in translating next-generation intervention paradigms into clinical applications, particularly for large, complex epigenetic editing systems [320]. Viral vectors, especially adeno-associated viruses (AAVs), have emerged as primary tools for in vivo gene therapy owing to their low immunogenicity, high safety profile, and tissue-targeting capabilities through various serotypes [321]. Nonetheless, their limited payload capacity restricts the delivery of large CRISPR-dCas9 effector proteins [320]. Accordingly, dual-AAV systems are being explored, including the use of smaller Cas proteins (e.g., SaCas9) and the engineering of the viral capsid to enhance tumor targeting and reduce immunogenicity [322–324]. In contrast, lentiviruses offer larger payload capacities and broader cell infection ranges, but the risk of insertional mutagenesis associated with their genomic integration remains a significant concern, driving the development of safer strategies such as non-integrating lentiviral vectors or lentiviral-like particles [325]. Nonviral vectors are also rapidly gaining traction. Lipid nanoparticles, which have been successfully used in COVID-19 mRNA vaccines, demonstrate scalability, low immunogenicity, and efficient nucleic acid delivery capabilities [326, 327]. Current research focuses on optimizing lipid components or conjugating targeting ligands to enhance tumor-targeting efficiency and promote endosomal escape [328]. Furthermore, exosomes, which are natural nanoparticles derived from cells, exhibit excellent biocompatibility and low immunogenicity, with the added advantage that their surfaces can be functionalized with targeting molecules through cell-source modifications [329]. Viral-like particles and engineered virus-derived nanoparticles combine the efficient transduction properties of viruses with enhanced safety [330]. For relatively small-molecule TPD drugs, delivery challenges are fewer than those for large molecular systems. However, their bifunctional structure often results in poor drug-like properties (e.g., low solubility and high polarity), which limit their oral bioavailability and membrane permeability [331, 332]. Developing prodrug strategies or utilizing nanocarriers for encapsulation are critical approaches for improving pharmacokinetic properties [333].

Ensuring that next-generation intervention paradigms are highly specific and safe—targeting only intended sites while avoiding off-target effects—is a core challenge in their clinical translation. In CRISPR-dCas9-based epigenetic editing systems, off-target effects mainly arise from mismatches between the gRNA and non-target sequences, potentially leading to unintended epigenetic modifications and cellular toxicity [334]. To address this, multiple optimization strategies are being developed concurrently: bioinformatics tools are used to design highly specific gRNA sequences, reducing off-target risks at the source [334]; high-fidelity dCas9 variants are engineered to enhance recognition accuracy [335]; and transient expression strategies (e.g., controlling the duration of dCas9 expression) are employed to limit the accumulation of off-target events [299]. However, the off-target effects of TPD drugs, exemplified by PROTACs, are complex. They may arise from ligand recognition of non-target proteins or from nonspecific ubiquitination of adjacent proteins induced by E3 ligases [336]. Enhancing selectivity involves developing high-specificity ligands and leveraging E3 ligases that are highly expressed in specific tissues or tumors [337, 338]. Notably, the selectivity of TPD depends not only on ligand–target protein affinity but also on the ability to form a stable, functional ternary complex [339].

The ability to precisely regulate the durability and reversibility of the therapeutic effects is a key consideration for the clinical translation of next-generation technologies. CRISPR/dCas9-based epigenetic editing (e.g., DNA methylation modifications) can produce heritable long-term effects [340]. Although this is beneficial for sustained efficacy, it also makes unintended edits difficult to correct [341]. Therefore, the development of inducible and reversible editing systems is critical to enhance safety and control [342]. In contrast, TPD exerts effects that are pharmacologically reversible. Target protein levels depend on the continued presence of the degrader, and once the drug is metabolized and cleared, the cell resynthesizes the target protein [343]. This controllability provides notable advantages for clinical dosage adjustment and toxicity management. Ongoing research is focused on developing controllable degraders with spatiotemporal or condition-responsive designs that enable more precise activity control [344], thereby improving therapeutic efficacy while minimizing toxicity to normal tissues [345]. This controllability is a key advantage of TPD over irreversible inhibitors [346]. Overall, epigenetic editing and TPD, through direct “reprogramming” of epigenetic information or “clearing” of regulatory proteins, go beyond traditional inhibition paradigms and offer revolutionary tools for the precise, efficient, and controlled reversal of drug resistance.

### Combined intervention: epigenetic therapies in conjunction with traditional and emerging treatments

In the context of reversing cancer treatment resistance, single epigenetic drugs frequently fail to address the heterogeneity of tumors and the dynamically evolving resistance networks [18]. As discussed in earlier sections, while epigenetic drugs have considerable theoretical potential for reprogramming cancer cell fate, their objective response rates as monotherapies in solid tumors have generally been modest [18]. An increasingly clear consensus has emerged that the true power of epigenetic interventions lies in their synergistic effects with other therapies. Through combination strategies, epigenetic therapies can cooperatively dismantle tumor defense mechanisms [228].

#### Reshaping tumor cell states to enhance sensitivity to direct cytotoxic therapies

Resistance to chemotherapy, radiotherapy, and targeted therapies often arises from complex epigenetic reprogramming [9]. For instance, aberrant hypermethylation of DNA promoter regions can silence key tumor suppressor, apoptosis-related, and drug-metabolizing genes, whereas specific histone modifications can activate pro-survival signaling pathways, enabling cancer cells to survive and proliferate under therapeutic stress [347]. Epigenetic drugs can fundamentally reset the sensitivity of cancer cells by reversing these dysregulated epigenetic marks, through a process known as epigenetic resensitization [8].

DNMTi, such as azacitidine and decitabine, exhibit strong synergistic effects in combination with traditional therapies through their demethylating actions [348]. Many key genes are frequently silenced by promoter hypermethylation, thereby facilitating drug resistance [3]. Preclinical studies have demonstrated that DNMTi can effectively reverse this silencing, restoring the expression of these genes and thereby resensitizing cancer cells to the corresponding chemotherapeutic agents, such as gemcitabine and platinum-based drugs [231, 348]. Disruption of DDR pathways to sensitize cells to chemotherapy and radiotherapy is a crucial synergistic mechanism. Platinum drugs and radiation primarily kill cancer cells by inducing DNA double-strand breaks, and tumor cells rely on efficient DDR systems, such as HR and non-homologous end-joining, to repair this damage. DNMTi have been reported not only to induce a degree of DNA damage [349] but, more importantly, to downregulate the expression of key DDR proteins, such as RECQ1, through demethylation [350]. This weakening of the DDR system causes cancer cells to accumulate irreparable DNA damage upon exposure to subsequent DNA-damaging agents or PARP inhibitors, ultimately leading to apoptosis [349, 350]. This strategy has been validated in preclinical models of AML, breast cancer, and prostate cancer, and has led to several clinical trials combining DNMTi with PARP inhibitors to target DDR-deficient tumors [349–351]. For example, in AML and breast cancer models, the combination of DNMTi and PARP inhibitors has been reported to markedly enhance therapeutic efficacy [349, 350].

Because of their core function in regulating chromatin structure and gene expression, HDACi have been extensively studied as therapeutic adjuvants. When combined with various direct cytotoxic therapies, HDACi enhance the antitumor efficacy in multiple dimensions and help overcome drug resistance [352]. In combination with chemotherapy, HDACi can regulate the expression of key cell cycle-related genes, arrest cancer cells in the G1 or G2/M phases, and render them more sensitive to cell cycle-specific chemotherapeutic agents [240]. Simultaneously, by modulating the expression of apoptosis-related genes, HDACi can lower the apoptotic threshold and promote chemotherapy-induced cell death [240]. In targeted therapy, HDACi have shown potential to reverse acquired resistance. For instance, in hematologic malignancies, HDACi, when combined with targeted drugs, can inhibit compensatory survival pathways and restore tumor cell sensitivity to targeted agents [353]. In EGFR-mutant non-small cell lung cancer, HDACi have been reported to suppress signaling pathway activation (e.g., by promoting EGFR degradation) and overcome resistance to EGFR inhibitors [260]. Furthermore, HDACi impair DDR, thereby enhancing the efficacy of platinum-based chemotherapy or PARP inhibitors [354].

Despite promising preclinical results, translating these combination strategies into clinical benefits remains challenging. The primary obstacles include the additive toxicity of combination therapies, determining the optimal dosing schedule (synchronous, sequential, or intermittent administration), and identifying patient populations most likely to benefit from such treatments [255, 260]. For example, while early clinical trials with DNMTi/HDACi in combination with chemotherapy have shown efficacy signals, toxicity such as anemia has limited their application [355]. Future research should focus on developing next-generation epigenetic drugs with greater specificity and reduced toxicity, and on exploring biomarker-driven precision combination therapy strategies.

#### Reshaping the tumor immune microenvironment to unleash the potential of immunotherapy

Recently, the combination of epigenetic therapies and ICIs has emerged as one of the most exciting strategies for cancer treatment [78]. Many solid tumors show little to no response to ICIs or develop acquired resistance, largely due to the immunosuppressive state of the tumor microenvironment, often referred to as “cold tumors” [356]. These tumors are characterized by low tumor antigen presentation, lack of effector T-cell infiltration, and an abundance of immunosuppressive cells, such as regulatory T cells (Tregs) and myeloid-derived suppressor cells (MDSCs) [356]. Epigenetic drugs are particularly suited to modulating multiple immune-related features of the tumor microenvironment, reversing immune suppression and facilitating the successful application of ICIs [78].

To be recognized by the immune system, tumor cells must effectively present unique tumor antigens. Epigenetic drugs enhance tumor antigenicity through several mechanisms. First, these drugs can upregulate antigen-presentation mechanisms (APMs). Tumor cells often evade immune recognition by epigenetically silencing the expression of major histocompatibility complex class I (MHC-I) molecules and their associated processing and presentation components (such as β2-microglobulin, TAP, and LMP). DNMTi and HDACi can reactivate the transcription of these APM genes through demethylation or by promoting histone acetylation, restoring MHC-I expression on the tumor cell surface and enhancing their capacity to present antigens to CD8^+^ T cells [357]. Additionally, DNMTi can induce a “viral mimicry” effect, thereby triggering type I interferon signaling [358]. As mentioned previously, this pathway bridges innate and adaptive immunity by directly inhibiting tumor growth and promoting immune-cell infiltration and activation, particularly T-cell reactivation, thus initiating a robust antitumor immune response [359]. Furthermore, epigenetic drugs can relieve epigenetic silencing of tumor-associated antigens, such as cancer-testis antigens, and induce their aberrant expression in tumor cells, thereby providing additional targets for recognition and attack by the immune system [360].

The regulatory effect of epigenetic drugs on ICIs, such as PD-L1, is context-dependent and exhibits complex and potentially bidirectional effects. HDACi can upregulate PD-L1 expression in certain tumors, which may initially appear to enhance immunosuppression. Conversely, this creates a potential target for anti–PD-1/PD-L1 therapies [230]. A PD-L1-negative “cold tumor” would not respond to ICIs; however, after pretreatment with epigenetic drugs such as HDACi, tumor cells express PD-L1, potentially transforming the tumor into an ICI-sensitive “hot” one. This suggests that epigenetic drugs can serve as preconditioning strategies for ICIs by co-targeting the PD-1/PD-L1 axis [230]. In contrast, next-generation epigenetic drugs, such as BETi, have shown potential to directly suppress PD-L1 expression. BET proteins such as BRD4 can bind to the super-enhancer region of CD274 and drive its transcription, whereas BETi can block this process, thereby reducing PD-L1 expression in tumor cells at the source. This provides another mechanistic basis for their combination with ICIs [361].

Epigenetic drugs can systematically reshape the infiltration and function of immune cells in the tumor immune microenvironment. One mechanism involves upregulating specific chemokines to recruit effector immune cells. In “cold tumors,” expression of key chemokines such as CXCL9 and CXCL10 is often suppressed by epigenetic modifications; DNMTi can relieve this suppression, thereby recruiting CXCR3-expressing CD8⁺ T cells and natural killer cells [362]. In addition, these drugs weaken the function of immunosuppressive cells. For example, epigenetic interventions, such as targeting the CXCR3 signaling pathway, can disrupt Treg stability and promote their conversion to a pro-inflammatory phenotype [363]. DNMTi have also been reported to reduce the recruitment and accumulation of MDSCs [364]. By weakening the suppressive capacity of these cell populations, the overall immune microenvironment shifts toward an antitumor phenotype [78]. Furthermore, epigenetic drugs can revitalize exhausted T cells. Tumor-infiltrating T cells often enter an “exhausted” state after prolonged engagement with tumor antigens, which is characterized by high expression of multiple inhibitory receptors such as PD-1, TIM-3, and LAG-3. BETi demonstrate unique advantages in this context [365]. BETi have been shown to directly act on T cells by epigenetically reprogramming them to downregulate inhibitory receptor expression, while promoting the survival and differentiation of progenitor-like T cells with self-renewal capacity, thereby restoring T-cell effector function [366, 367].

Building on a solid theoretical foundation, the combination of epigenetic therapies and ICIs has made considerable progress in several clinical trials. For example, in the ENCORE-601 phase II trial targeting PD-1 inhibitor resistance in patients with metastatic melanoma, a combination including the HDACi entinostat achieved an objective response rate of 28.6% [368]. In other cancer types, such as bladder cancer, combination therapies using DNMTi and ICIs have shown preliminary efficacy and safety [56, 369]. By the end of 2025, hundreds of related combination therapy clinical trials were ongoing worldwide (Table 3).

**Table 3:** Clinical strategies for the combination of epigenetic drugs and other drugs (2020‒2025) (clinicaltrials.gov)

Type	Drug	Combination drug	Cancer type	Status	Phase	NCT number
DNMTi	CC-486	Romidepsin+Lenalidomide+Dexamethasone	T-cell malignancies	Active, not recruiting	I	NCT04447027
	Azacitidine	Enasidenib+Ivosidenib+Venetoclax	Acute myeloid leukemia	Recruiting	II	NCT05401097
BETi	ZEN003694	Niraparib	Solid tumors	Withdrawn	I	NCT06161493
	ZEN003694	Enzalutamide	Prostate cancer	Recruiting	II	NCT04986423
	ZEN-3694	Enzalutamide+Pembrolizumab	Prostate cancer	Active, not recruiting	II	NCT04471974
	ZEN-3694	Talazoparib	Solid tumors	Recruiting	II	NCT05327010
	ZEN003694	Abemaciclib	Breast cancer and other solid tumors	Recruiting	I	NCT05372640
	ZEN003694	Entinostat	Solid tumors and lymphomas	Recruiting	I/II	NCT05053971
	ZEN003694	Binimetinib	Solid tumors and triple-negative breast cancer	Suspended	I	NCT05111561
	ZEN003694	Capecitabine	Metastatic or unresectable cancers	Recruiting	I	NCT05803382
	ZEN003694	Nivolumab	Solid tumors	Recruiting	I	NCT04840589
	ZEN003694	Tuvusertib	Recurrent ovarian and endometrial cancer	Recruiting	I	NCT05950464
	ZEN003694	Cetuximab+Encorafenib	Colorectal cancer	Recruiting	I	NCT06102902
	ZEN003694	Pembrolizumab+Nab-paclitaxel	Triple-negative breast cancer	Recruiting	I	NCT05422794
	ZEN003694	Cisplatin+Etoposide	NUT carcinoma	Recruiting	I/II	NCT05019716
HDACi	Enitinostat	Dalpiciclib	Breast cancer	Not yet recruiting	II	NCT06556862
	ABT-301	Tislelizumab+Bevacizumab	Colorectal cancer	Recruiting	I/II	NCT07244705
	Seclidemstat	Pembrolizumab	Endometrioid ovarian cancer + endometrioid endometrial cancer	Withdrawn	I	NCT04611139
	CC-90011	Abiraterone+Prednisone	Prostate cancer	Completed	I	NCT04628988
	CC-90011	Nivolumab	Lung cancer	Completed	II	NCT04350463
	CC-90011	Venetoclax+Azacitidine	Acute myeloid leukemia	Terminated	I	NCT04748848
	IMG-7289	Venetoclax	Acute myeloid leukemia	Suspended	I	NCT05597306
	Seclidemstat	Azacitidine	Myelodysplastic syndrome + chronic myelomonocytic leukemia	Recruiting	I/II	NCT04734990
	Iadademstat	Azacitidine+Venetoclax	Acute myeloid leukemia	Recruiting	I	NCT06357182
	Bomedemstat	Atezolizumab	Extensive stage small cell lung cancer	Terminated	I/II	NCT05191797
	SHR2554	HRS-8080+HRS-1358	Breast cancer	Not yet recruiting	II	NCT07125950
	SHR2554	SHR-A2102+Adabelimumab+SHR-1701	Non-small cell lung cancer	Recruiting	II	NCT07175220
	SHR2554	SHR-0302	T-cell lymphoma	Recruiting	II	NCT06519526
	Romidepsin	Durvalumab	Bladder cancer	Recruiting	I	NCT06963346
	Romidepsin	Parsaclisib	T-cell lymphoma	Completed	I	NCT04774068
	Romidepsin	Copanlisib	T-cell lymphoma	Withdrawn	I	NCT04233697
	Chidamide	Toripalimab	Sarcoma	Recruiting	II	NCT04025931

#### Rational combinations and AI-driven personalized therapy

As our understanding of the complexity of epigenetic regulatory networks continues to evolve, cancer treatment strategies are transitioning from single-target interventions to multitarget, multidimensional approaches, ultimately progressing toward personalized precision medicine.

Epigenetic regulation does not occur in isolation but involves complex interactions across multiple dimensions. These findings provide a theoretical basis for combining different epigenetic pathways as therapeutic targets [370]. Nevertheless, targeting a single pathway may be insufficient to fully reverse abnormal epigenetic states. Thus, combining different classes of epigenetic drugs, with the goal of achieving synergistic effects where “1 + 1 > 2,” has emerged as a rational direction for therapeutic development [228]. Among these, drug combinations targeting distinct epigenetic mechanisms, such as DNA methylation and histone modifications, are the primary focus of research because they can more thoroughly reactivate TSGs or inhibit oncogenic pathways through synergistic effects [18]. Such combination therapies have shown superior efficacy compared with monotherapy in hematologic malignancies, such as leukemia, and are increasingly being explored for solid tumors [18, 78]. In addition, as novel drugs enter clinical trials, progressively complex dual-inhibition strategies are emerging. For example, combining inhibitors of histone methyltransferases (such as G9a) and DNMT1 enables the coordinated regulation of histone and DNA methylation, simultaneously targeting both tumor suppressor and oncogenic pathways to achieve more effective antitumor activity [262, 371].

The ultimate goal is to tailor the optimal combination regimen for each patient; meeting the challenge of profound intratumoural heterogeneity will require integrated multi-omics profiling coupled with artificial intelligence (AI) [372]. Conventional precision oncology often relies on single biomarkers (e.g., EGFR mutations), whereas the complexity of epigenetic regulation demands broader and more integrated molecular signatures to guide therapy selection [373]. By combining genomics (to identify alterations in epigenetic modifiers), epigenomics (to map DNA methylation, chromatin accessibility, and histone modifications using approaches such as whole-genome bisulfite sequencing, assay for transposase-accessible chromatin sequencing, and chromatin immunoprecipitation sequencing), transcriptomics (RNA sequencing–based expression profiling), and proteomics, it is possible to obtain a comprehensive view of an individual tumor’s epigenetic state, pathway activity, and immune microenvironment architecture [372, 374].

AI offers a crucial framework for extracting actionable patterns from high-dimensional data. Models trained on multi-omics datasets and treatment-response annotations from large clinical trials and preclinical studies can learn the associations between specific molecular features and sensitivity or resistance to defined combination strategies (e.g., DNMTi + anti–PD-1 or EZH2i + ICIs) [375]. For a new patient, AI can leverage the multi-omics profile of the tumor to assign it to predefined subtypes with characteristic biological features and response trajectories. For example, a model may identify a subtype marked by epigenetically silenced immune programs and sparse immune-cell infiltration [376]. Guided by this classification, AI systems can estimate the probability of response across candidate regimens and prioritize the most promising option, such as epigenetic therapy combined with ICIs, for an epigenetically silenced subtype [375].

Despite the potential of AI-driven, multi-omics-based personalized epigenetic therapies, their routine clinical application remains in its infancy. Currently, there is a lack of large-scale prospective clinical trials demonstrating substantial improvements in patient outcomes associated with AI-recommended epigenetic combination therapies. Several challenges continue to limit clinical translation, including the need for standardization of multi-omics platforms, high testing costs, difficulties in generating robust training datasets, limited interpretability of AI models (the “black box” problem), and obstacles to integrating computational tools in clinical settings [377]. Currently, clinical trials focused on personalized epigenetic combination therapies remain largely exploratory, and no large-scale studies with publicly reported results have yet been completed [378].

**Fig. 4:**
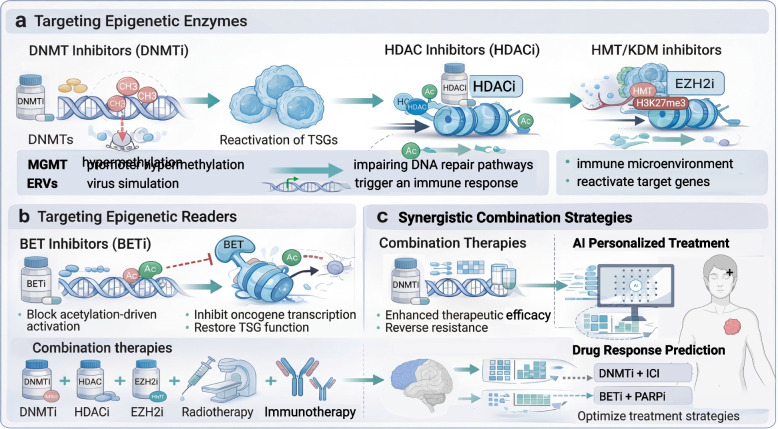
Strategies to target the epigenetic landscape to overcome therapy resistance. **a** Targeting epigenetic enzymes. The schematic illustrates how inhibitors (e.g., DNMTi, HDACi, EZH2i) reactivate antitumor programs by reversing the epigenetic silencing of tumor suppressor genes. DNMT inhibitors (DNMTi) exert their effects through multiple mechanisms, including reactivation of silenced tumor suppressor genes, disruption of DNA repair pathways, and induction of immunogenic viral mimicry, collectively resensitizing tumors to therapy. EZH2 inhibitors overcome resistance by reactivating repressed target genes and reprogramming the tumor immune microenvironment. **b** Targeting epigenetic readers: BET inhibitors (BETi) block acetylation-driven transcription activation, restoring TSG function and inhibiting oncogene expression. **c **Synergistic combination strategies: Combination therapies, such as DNMTi with immune checkpoint inhibitors (ICIs), enhance therapeutic efficacy and reverse resistance; AI-guided personalized approaches predict drug responses and optimize treatment strategies, advancing precision in clinical applications

In the future, clinical trials must be structured around molecular features and epigenetic biomarkers, enabling patients to be allocated to tailored epigenetic combination therapies based on their individual multi-omics characteristics [379]. With advances in technology and cost reduction, the integration of AI and multi-omics will undoubtedly become a core driver of synergistic epigenetic interventions and of overcoming cancer drug resistance, leading to a new era of precision cancer therapy (Fig. 4).

## Discussion and perspectives

In this comprehensive analysis of the role of epigenetic regulation in cancer treatment resistance, we provide insight into the complexity and depth of this regulatory network. Tumor resistance does not arise from a single, fixed molecular event, but rather emerges from an epigenetic landscape shaped by interconnected layers, including DNA methylation, histone modifications, chromatin remodeling, and non-coding RNAs [4]. This ecosystem bestows tumor cells with remarkable adaptability, enabling them to flexibly switch cell states, activate bypass signals, and reshape the immune microenvironment under therapeutic pressure, thereby creating a formidable barrier to treatment [9]. However, it is precisely the core features of this system (plasticity and reversibility) that offer unprecedented therapeutic opportunities [347].

Translating this mechanistic insight into clinical benefit requires confronting key limitations of current approaches. Most epigenetic drugs (e.g., DNMTi/HDACi) remain rooted in a static target paradigm, broadly inhibiting “writers” or “erasers” to reverse global silencing. However, this broad-spectrum approach fails to reflect resistance as a coordinated and dynamically evolving process of network adaptation [380]. In complex epigenetic networks, single-enzyme inhibition can produce unpredictable effects and readily trigger compensatory reprogramming, occasionally reinforcing resistant states [381]. Further progress may involve a shift toward dynamic network regulation, integrating single-cell multi-omics with computational modeling to identify regulatory nodes and circuits involved in sustaining resistance [382]. Such strategies could guide the development of precise tools, such as allosteric modulators, protein interaction molecules, and epigenetic editors, to reset dysregulated states and restore treatment sensitivity, rather than relying on non-selective cytotoxicity [1].

Without actionable delivery, epigenetic intervention strategies remain largely underutilized. Systemic dosing of epigenetic drugs is still limited by off-target toxicity and poor penetration into solid tumors, thereby narrowing the therapeutic window [296]. A credible path forward is cell-selective, spatiotemporally programmable delivery: carriers that actively recognize drug-resistant surface markers and execute multi-cue release (e.g., pH- and enzyme-responsive mechanisms) to restrict exposure to the intended cellular niche [383]. Efficient nuclear delivery of precision tools such as CRISPR-based epigenetic editors and PROTAC degraders would enable “ecological niche editing,” preferentially eliminating resistant subpopulations while sparing normal tissues [384].

Likewise, the clinical evaluation of epigenetic reprogramming therapies must move beyond static biomarkers and conventional endpoints developed for cytotoxic agents. Translation depends on dynamic evaluation and decision frameworks aligned with the time-varying nature of epigenetic state transitions [385, 386]. This includes adaptive trial designs in which treatment can be modified in response to on-therapy biomarker trajectories, longitudinal monitoring enabled by liquid biopsy to noninvasively track the evolution of epigenetic networks [387], and the integration of multi-omics time-series data using artificial intelligence to build predictive models that guide individualized interventions. Collectively, these components would enable a shift from population-level empiricism to data-driven, prospective, and adaptive therapy.

Cancer therapy stands at an inflection point. Over the past century, the prevailing strategy has been “maximal cytotoxicity,” but the plasticity of tumor epigenetic systems suggests that indiscriminate cell killing may drive the emergence of increasingly adaptable, drug-resistant populations. Therefore, we propose three paradigm shifts: from static target intervention to dynamic network regulation; second, from broad-spectrum exposure to precise delivery; and third, from empirical combinations to algorithm-guided personalization. Taken together, these shifts advance a new vision that replaces “maximal cytotoxicity” with “precise ecological regulation”.In this vision, the goal is not necessarily complete eradication, but rather the reprogramming of a malignant, unstable tumor ecosystem into a benign, stable state compatible with long-term containment and coexistence with the host. This approach reconceptualizes cancer therapy, shifting the focus from aggressive eradication to sustainable long-term control, and provides a rationale foundation for future therapeutic progress.

## Data Availability

No datasets were generated or analyzed during the current study.

## Abbreviations

DNMTs: DNA methyltransferases
HDACs: Histone deacetylases
HATs: Histone acetyltransferases
HMTs: Histone methyltransferases
KDMs: Lysine demethylases
BET: Bromodomain and extraterminal
miRNA: MicroRNA
UTR: Untranslated region
RISC: RNA-induced silencing complex
lncRNA: Long non-coding RNA
PRC2: Polycomb repressive complex 2
EZH2: Enhancer of zeste homolog 2
TSG: Tumor suppressor gene
RASSF1A: Ras association domain family member 1A
PTEN: Phosphatase and tensin homolog
mTOR: Mechanistic target of rapamycin
MAPK: Mitogen-activated protein kinase
HER2: Human epidermal growth factor receptor 2
ERK: Extracellular signal-regulated kinase
MET: Mesenchymal-epithelial transition factor
EGFR: Epidermal growth factor receptor
RTKs: Receptor tyrosine kinases
CSCs: Cancer stem cells
LSCs: Leukemia stem cells
Sox9: SRY-box transcription factor 9
EMT: Epithelial-mesenchymal transition
LSD1: Lysine-specific demethylase 1
DTPs: Drug-tolerant persister cells
ABC: ATP-binding cassette
P-gp: P-glycoprotein
BCRP: Breast cancer resistance protein
SLC: Solute carrier
CYP: Cytochrome P450
TAMs: Tumor-associated macrophages
IL: Interleukin
JMJD3: Jumonji domain-containing protein 3
TOX: Thymocyte selection-associated high mobility group box
PD-1: Programmed cell death protein 1
CAFs: Cancer-associated fibroblasts
DNMTi: DNMT inhibitor
ERVs: Endogenous retroviral elements
MAVS: Mitochondrial antiviral-signaling protein
HDACi: HDAC inhibitor
HSP90: Heat shock protein 90
NSCLC: Non-small cell lung cancer
BETi: BET inhibitor
BCL-2: B-cell lymphoma 2
ENL: Eleven-nineteen leukemia
MLL: Mixed-lineage leukemia
DOT1L: Disruptor of telomeric silencing 1-like
NuRD: Nucleosome remodeling and deacetylase
RNAi: RNA interference
MEK: Mitogen-activated protein kinase kinase
dCas9: Catalytically dead Cas9
sgRNA: Single guide RNA
CRISPRa: CRISPR activation
CRISPRi: CRISPR interference
KRAB: Krüppel-associated box
CRISPRoff: CRISPR off
CRISPRon: CRISPR on
PROTACs: Proteolysis-targeting chimeras
POI: Protein of interest
AAV: Adeno-associated virus
SaCas9: *Staphylococcus aureus* Cas9
TPD: Targeted protein degradation
CXCL: C-X-C motif chemokine ligand
NK cells: Natural killer cells
Tregs: Regulatory T cells
MDSCs: Myeloid-derived suppressor cells
ICIs: Immune checkpoint inhibitors
G9a: Euchromatic histone lysine methyltransferase 2
AI: Artificial intelligence
WGBS: Whole-genome bisulfite sequencing
ATAC-seq: Assay for transposase-accessible chromatin sequencing
ChIP-seq: Chromatin immunoprecipitation sequencing
RNA-seq: RNA sequencing
MDR: Multidrug resistance
DSBs: DNA double-strand breaks
NHEJ: Non-homologous end-joining
HR: Homologous recombination
FGFR: Fibroblast growth factor receptor
ZEB: Zinc finger E-box binding homeobox
TWIST: Twist family bHLH transcription factor
SNAIL: Snail family transcriptional repressor
SLC31A1: Solute carrier family 31 member 1
JMJD3: Jumonji domain-containing protein 3
TET: Ten-eleven translocation
